# Discovery of a novel third-generation EGFR inhibitor and identification of a potential combination strategy to overcome resistance

**DOI:** 10.1186/s12943-020-01202-9

**Published:** 2020-05-13

**Authors:** Tao Zhang, Rong Qu, Shingpan Chan, Mengzhen Lai, Linjiang Tong, Fang Feng, Hongyu Chen, Tingting Song, Peiran Song, Gang Bai, Yingqiang Liu, Yanan Wang, Yan Li, Yi Su, Yanyan Shen, Yiming Sun, Yi Chen, Meiyu Geng, Ke Ding, Jian Ding, Hua Xie

**Affiliations:** 1 Division of Antitumor Pharmacology, State Key Laboratory of Drug Research, Shanghai Institute of Materia Medica, Chinese Academy of Sciences, 555 Zuchongzhi Road, Shanghai, 201203 China; 2 International Cooperative Laboratory of Traditional Chinese Medicine Modernization and Innovative Drug Development of Chinese Ministry of Education (MOE), Guangzhou City Key Laboratory of Precision Chemistry Drug Development, School of Pharmacy, Jinan University, No. 601 Huangpu Avenue West, Guangzhou, 510632 China; 3 School of Pharmacy, Fudan University, 826 Zhangheng Road, Shanghai, 201203 China; 4 Jiangsu Aosaikang Pharmaceutical Co.Ltd (ASK pharm), 699 Kejian Road, Nanjing, 211112 China; 5 University of Chinese Academy of Sciences, 19A Yuquan Road, Beijing, 100049 China; 6 School of Life Science and Technology, ShanghaiTech University, 393 Middle Huaxia Road, Shanghai, 201210 China

**Keywords:** Non-small cell lung cancer (NSCLC), EGFR T790M, Small-molecule inhibitor, Drug resistance, Ack1

## Abstract

**Background:**

Non-small cell lung cancer (NSCLC) patients with activating EGFR mutations initially respond to first-generation EGFR inhibitors; however, the efficacy of these drugs is limited by acquired resistance driven by the EGFR ^*T*790*M*^ mutation. The discovery of third-generation EGFR inhibitors overcoming EGFR ^*T*790*M*^ and their new resistance mechanisms have attracted much attention.

**Methods:**

We examined the antitumor activities and potential resistance mechanism of a novel EGFR third-generation inhibitor in vitro and in vivo using ELISA, SRB assay, immunoblotting, flow cytometric analysis, kinase array, qRT-PCR and tumor xenograft models. The clinical effect on a patient was evaluated by computed tomography scan.

**Results:**

We identified compound ASK120067 as a novel inhibitor of EGFR ^*T*790*M*^, with selectivity over EGFR ^*WT*^. ASK120067 exhibited potent anti-proliferation activity in tumor cells harboring EGFR ^*T*790*M*^ (NCI-H1975) and sensitizing mutations (PC-9 and HCC827) while showed moderate or weak inhibition in cells expressing EGFR ^*WT*^. Oral administration of ASK120067 induced tumor regression in NSCLC xenograft models and in a PDX model harboring EGFR ^*T*790*M*^. The treatment of one patient with advanced EGFR T790M-positive NSCLC was described as proof of principle. Moreover, we found that hyperphosphorylation of Ack1 and the subsequent activation of antiapoptotic signaling via the AKT pathway contributed to ASK120067 resistance. Concomitant targeting of EGFR and Ack1 effectively overrode the acquired resistance of ASK120067 both in vitro and in vivo.

**Conclusions:**

Our results idenfity ASK120067 as a promising third-generation EGFR inhibitor and reveal for the first time that Ack1 activation as a novel resistance mechanism to EGFR inhibitors that guide to potential combination strategy.

## Backgroud

Abnormal activation of epidermal growth factor receptor (EGFR) is associated with a variety of tumors, especially non-small cell lung cancer (NSCLC) [1–3]. Tumors containing activating EGFR mutations, such as deletion in exon 19 (19del) and the L858R point mutation, account for approximately 10–15% of NSCLC cases in individuals of European descent and 30% of NSCLC cases in individuals of East Asian descent [4]. Although these mutations in EGFR are sensitive to inhibition by established tyrosine kinase inhibitor (TKI) therapies, such as gefitinib, erlotinib and icotinib, almost all tumors eventually develop acquired resistance to these TKIs within 9–14 months [3, 5]. The most common mechanism of TKI resistance is a second-site mutation (T790M) in the EGFR kinase domain, which accounts for more than 50% of these cases [6]. Mutant-selective third-generation EGFR TKIs have been developed with the goal of overcoming T790M-mediated resistance. Osimertinib (AZD9291) is the only third-generation TKI approved by the FDA to date for the treatment of patients with advanced EGFR T790M mutation-positive NSCLC [7]. Clinical studies have demonstrated that osimertinib monotherapy induces an approximately 60% response rate and a durable benefit in patients with lung cancers harboring the EGFR T790M mutation [8, 9]. Encouraged by the success of osimertinib, researchers have designed many other selective inhibitors against EGFR T790M that are now at different stages of clinical or preclinical studies [10–14].

Although third-generation EGFR TKIs have proven effective in patients with NSCLC who have developed the T790M mutation, the majority of patients will develop resistance to these drugs and undergo disease progression. Acquired mutations in EGFR, such as C797S [15, 16], L792F/H [17, 18] and L718Q [19], have been discovered as clinically relevant resistance mechanisms to osimertinib. In addition, the activation of signaling molecules, including MET [20], BRAF [21], PIK3CA [22, 23], AXL [24], and Src family kinases (SFK) [25], to bypass osimertinib-mediated activity as well as the induction of epithelial-to-mesenchymal transition (EMT) [12, 26], also largely contributed to the acquired resistance to third-generation EGFR TKIs. Notably, no mechanism of resistance to third-generation EGFR TKIs has been identified in up to half of all tumors reported to date, and the remaining resistance mechanisms to this TKI are largely unknown. The heterogeneous nature of acquired resistance motivates a broad search for additional resistance mediators that may provide opportunities for novel therapeutic approaches.

Ack1 (activated Cdc42-associated tyrosine kinase 1), a non-receptor tyrosine kinase (non-RTK), is an important signaling molecule involved in integrating signals from a number of different receptor tyrosine kinases, such as EGFR, anaplastic lymphoma kinase (ALK), AXL and insulin receptor (IR) [27, 28]. It can transduce extracellular signals to cytosolic and nuclear effectors, distinguishing it from many other non-RTKs. Overexpression, amplification, or mutation of Ack1 was positively correlated with the severity of disease progression and inversely correlated with the survival of cancer patients. Activation of Ack1 occurs in multiple cancers, such as primary endocrine and hormone-driven tumors. These cancers display increased Ack1 activation by modulating Ack1 gene expression at the transcriptional level [29–34]. Interestingly, studies in recent years have revealed that Ack1 is involved in the resistance of hormone-dependent tumors. Mahajan and colleagues reported that Ack1 can interact with the estrogen receptor (ER)/histone demethylase KDM3A (JHDM2a) complex and promote the growth of tamoxifen-resistant breast cancer through epigenetic regulation [35]. In addition, Ack1 has also been shown to be critical for the progression of prostate cancer to the hormone therapy-insensitive stage, termed castration-resistant prostate cancer (CRPC) due to its ability to regulate the expression and function of androgen (AR) in an AR-independent manner [36, 37]. In addition to increased kinase activation in breast and prostate cancers, Ack1 gene amplification is a frequent event in NSCLC. Pharmacological inhibition of Ack1 attenuates migration and invasion in the context of KRAS-mutant NSCLC [38]. Whether Ack1 activation is involved in the drug resistance of NSCLC patients remains unclear.

In the current study, we report the identification and characterization of a novel third-generation EGFR TKI, ASK120067, that irreversibly and selectively inhibits mutant EGFR in NSCLC models. Oral administration of ASK120067 leads to tumor regression in cell-based and patient-derived xenograft (PDX) models harboring EGFR mutants. Moreover, acquired resistance to this compound was also investigated. Hyperactivation of Ack1 was found to be associated with resistance to ASK120067, and the combination of ASK120067 with Ack1 inhibitors showed a synergistic effect in ASK120067-resistant cells both in vitro and in vivo. ASK120067 is currently being evaluated in phase I/II clinical trials in EGFR-mutant NSCLC in China.

## Materials and methods

### The enzyme-linked immunosorbent assay (ELISA)-based kinase activity assay

EGFR ^*exon*19*del*^ (#PV6179) protein was purchased from Life. EGFR ^*L*858*R*/*T*790*M*^ (#14-721MM), EGFR ^*T*790*M*^ (#14-725), EGFR ^*WT*^ (#14-531M) were purchased from Eurofins. The kinase activities were evaluated with ELISA according to previously described protocols [39].

### Cell culture and compound reagents

NCI-H1975, PC-9, HCC-827, A431, LoVo and A549 cell lines were obtained from the American Type Culture Collection (ATCC). All cells were authenticated by short tandem repeat (STR) analysis performed by Genesky.

### In vitro cell proliferation assays

The inhibitory activity of compounds on growth was evaluated using the sulforhodamine B (SRB) colorimetric assay. Cells were seeded in 96-well plates, cultured overnight, and treated with a dilution series of test compounds for 72 h. Then, the SRB assay was performed according to standard protocols, as described previously [40].

### Immunoblotting analysis

Cells were lysed in SDS lysis buffer. After heating for 15 min at 100 ^∘^C, whole cell lysis samples were loaded onto SDS-PAGE gels, followed by transfer to nitrocellulose membranes. Membranes were blocked with 5% milk-TBST and then blotted with primary antibodies against phospho-EGFR (Tyr1068;#3777), EGFR (#4267), phospho-ERK (T202/Y204; #4370), ERK1/2 (#4695), phospho-AKT (Ser473; #4060), pan-AKT (#4691), caspase-3 (#9662S), cleaved caspase-3 (Asp175) (#9664S), PARP (#9532S), BIM (#2933S), *β*-tubulin (#2146) (all from Cell Signaling Technology); p-Ack1 (phosphoY284; Abcam, #ab74091); Ack1 (Santa Cruz, #sc-28336), *β*-actin (Abgent, #P60709) and GAPDH (Proteintech, #60004-1-lg) followed by treatment with horseradish peroxidase (HRP)-conjugated secondary antibodies (Jackson, #111-035-003).

Tumor tissues were lysed with RIPA supplemented with protease inhibitor cocktail and phosphatase inhibitor (Roche). Protein concentrations were determined using a standard Bradford assay (Beyotime) for normalization of the samples. Equal amounts of protein were loaded on SDS-PAGE gels for blotting.

### Apoptosis assays

Apoptosis of tumor cell lines was measured by Annexin V and propidium iodide (PI) dual staining using an Annexin V-FITC Apoptosis Detection Kit (Vazyme, #A211-02). Signals were detected using a FACS Calibur flow cytometer (Becton Dickinson).

Apoptosis in tumor tissues was measured by TUNEL (terminal deoxynucleotidyl transferase (TdT)-mediated dUTP nick end labeling) staining using an In Situ Cell Death Detection Kit, POD (Roche, #11684817910). Assays were performed according to standard protocols provided by Shanghai ZuoChengBio.

### In vivo antitumor efficacy study

Tumor cells (5×10^6^) were subcutaneously injected into the right flanks of BALB/cA nude mice. After one passage, well-developed tumors were chopped into 1.5 mm^3^ fragments and transplanted into the right flanks of nude mice using a trocar. When the tumor volume reached 100–200 mm^3^, mice were randomly assigned into vehicle and treatment groups and dosed with vehicle control or test compounds, respectively. The PDX xenograft study was performed by CrownBio. Tumor tissues from HuPrime ^*Ⓡ*^ patient-derived xenograft (LU1868, EGFR^*L858R*/*T790M*^) were subcutaneously injected into the right flanks of BALB/cA nude mice. When the tumor volume reached 150–200 mm^3^, mice were randomly assigned into vehicle and treatment groups and dosed with vehicle control or ASK120067, respectively. Tumor growth was monitored twice per week. All procedures related to animal care, handling, and treatment were performed according to the guidelines approved by the Institutional Animal Care and Use Committee following the guidance of Association for Assessment and Accreditation of Laboratory Animal Care.

### Immunohistochemistry

Paraffin-embedded tumor tissue sections underwent antigen retrieval, endogenous peroxidase blocking and primary antibody incubation overnight at 4 ^∘^C. Slides were rinsed, incubated with enhancer for 20 min, rinsed again, and then treated with peroxidase-labeled polymer for 20 min at 37 ^∘^C. Sections were stained with DAB and counterstained with hematoxylin. Images were captured on an inverted microscope (Olympus BX51). Quantitative analysis was performed with Image-Pro Plus 6. Primary antibodies against phospho-EGFR (Y1173) (#4407S), phospho-AKT (Ser473) (#4060) and Ki-67 (#9449S) were purchased from Cell Signaling Technology, and primary antibodies against p-Ack1 (phosphoY284; bs-3045R) were purchased from Bioss. The peroxidase-based immunohistochemistry detection kit (#PV-9001), 3,3’-diaminobenzidine (DAB) (#ZLI-9018), antigen retrieval buffer (pH 9.0) (#ZLI-9069) and antibody diluent (#ZLI-9028) were obtained from ZSbio.

### Generation of cells resistant to ASK120067 or osimertinib

ASK120067-resistant cells (67R) or osimertinib-resistant cells (AZDR) were obtained from parental NCI-H1975 cells by a dose-escalation method [25]. NCI-H1975 cells were treated with increasing doses of ASK120067 or osimertinib at 5 nM, followed by a stepwise dose escalation every 2–3 generations up to 1 *μ*M, and then the resistant cells (67R or AZDR) were maintained in 1 *μ*M corresponding compounds.

### Genetic analysis

NCI-H1975, 67R and AZDR in the logarithmic growth period were collected with 10^7^ cells per sample. The DNA quality of the sample was tested by real-time fluorescence quantitative PCR, and whole genome sequencing (WGS) of NCI-H1975, 67R and AZDR cells as well as the analysis of the sequencing data were performed by Wuxi PharmaTech.

### Quantitative RT-PCR

Cells were treated with DMSO or the indicated compounds for 48 h before being subjected to RNA purification via an EZ-press Cell to cDNA Kit (EZBioscience, #B0001). Samples were then analyzed for mRNA expression via qRT-PCR using the iTaq ^TM^ Universal SYBR *Ⓡ* Green Supermix (BioRad, #1725125) and 7500 real-time PCR instrument (Applied Biosystems). The primer sequences were as follows: BIM, forward primer, 5’-TGGGTATGCCTGCCACATTTC-3’, reverse primer, 5’-CCACGTTTTTGACGATGGAGA-3’; GAPDH, forward primer, 5’-CCACCCATGGCAAATTCCATGGCA-3’, reverse primer, 5’-TCTAGACGGCAGGTCAGGTCCACC-3’. Primer synthesis was completed by Sango Biotech.

### Statistical analysis

All experiments were repeated at least three times, and the data are presented as mean ± standard deviation (SD) or mean ± standard error of mean (SEM). The statistical analyses were performed using GraphPad Prism. Difference between two groups were analyzed by Student’s *t* test (two-sided) and significance was set at *p* < 0.05.The specific details about statistical methods are introduced in respective figure legends.

## Results

### ASK120067 is an irreversible third-generation EGFR inhibitor that selectively targets the T790M-resistant mutant and sensitizing mutants

**Fig. 1:**
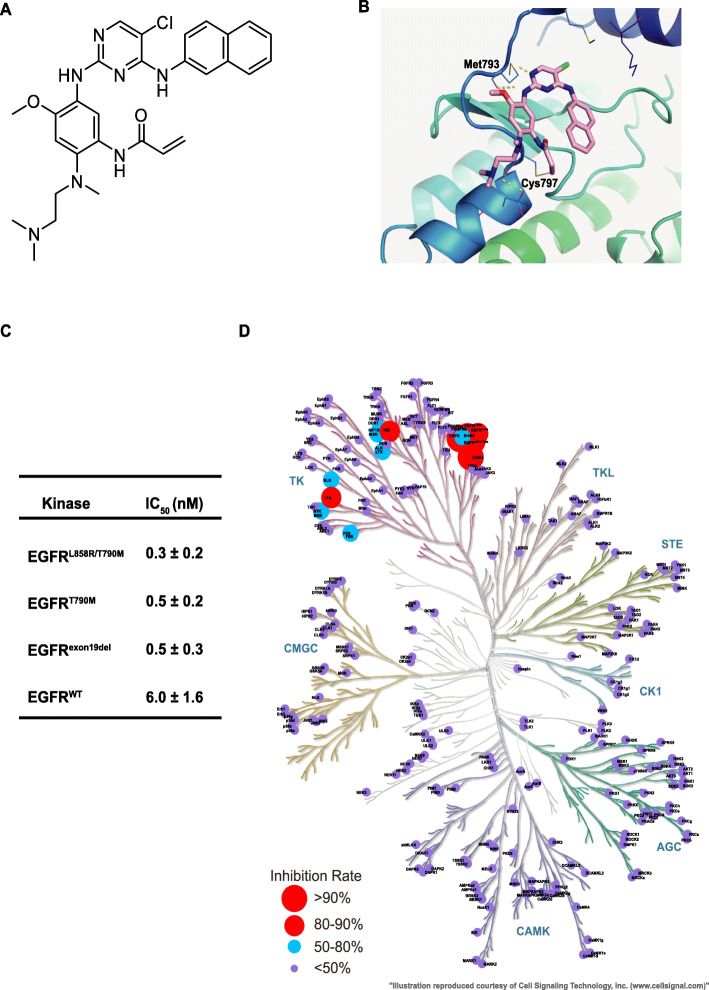
Chemical structure, binding mode and target inhibition of compound ASK120067. **a** Chemical structure of ASK120067. **b** Structure modeling of ASK120067 binding to EGFR ^*T*790*M*^. The 2-aminopyrimidine core of ASK120067 forms two hydrogen bonds to the hinge residue Met793, and the acrylamide group forms a covalent bond with the conserved C797 residue in the ATP-binding pocket. The C5-Cl substitution points to the gatekeeper Met790 residue. The 2,4-disubstituted pyrimidine scaffold adapts a U-shaped configuration. The amine moiety faces an open space in the solvent exposure area. **c** Inhibition of ASK120067 on EGFR kinases determined by ELISA assay. IC_50_ values are shown as the mean ± SD from at least three independent experiments. **d** Kinase profiling of ASK120067 at a concentration of 100 nM against 258 human kinases. The graphic was generated by BioMed X GmbH and Merck KgaA, with slight modifications. Circles indicate detected kinases, and the circle size indicates the strength of inhibition

Using a structure-based approach, we rationally designed and developed a series of novel molecules to target sensitizing and T790M-mutant resistant forms of EGFR with selectivity over wild-type EGFR. Among them, ASK120067 was identified as a distinct molecule (Fig. 1a). As modeling of this compound in complex with EGFR ^*T*790*M*^ protein showed that (PDB: 3IKA, Fig. 1b), the 2-aminopyrimidine core of ASK120067 forms two hydrogen bonds to the hinge residue Met793, while the acrylamide group forms the covalent bond to conserved cysteine-797 residue in the ATP-binding pocket. The C5-Cl substitution points to gatekeeper Met790 residue. 2,4-disubstituted pyrimidine scaffold adapt a U-shaped mode. The amine moiety faces an open space in the solvent exposure area.

The in vitro kinase assay confirmed that ASK120067 potently inhibited the EGFR ^*L*858*R*/*T*790*M*^ and EGFR ^*T*790*M*^ resistant mutants, with half maximal inhibitory concentrations (IC_50_) of 0.3 nM and 0.5 nM, respectively, as well as the EGFR ^*exon*19*del*^ sensitizing mutant (IC_50_= 0.5 nM). The IC_50_ of ASK120067 against wild-type EGFR (EGFR^*WT*^) was 6 nM, indicating a 20 times greater potency against EGFR ^*L*858*R*/*T*790*M*^ than against EGFR ^*WT*^ (Fig. 1c). To determine the selectivity of ASK120067, we profiled ASK120067 against a panel of 258 kinases using a Kinase Profiler platform, and ASK120067 exhibited a favorable selectivity profile (Fig. 1d).

### ASK120067 selectively inhibits the growth of EGFR-mutant cell lines and induces apoptosis

The activity and selectivity of ASK120067 against cells expressing EGFR mutations was assessed in a panel of cell lines, including NSCLC cell lines harboring either the EGFR ^*L*858*R*/*T*790*M*^ double mutation (NCI-H1975 cells) or EGFR ^*exon*19*del*^ (PC-9 and HCC827 cells) and three cell lines expressing wild-type EGFR (A431, LoVo and A549). ASK120067 exhibited potent antiproliferative activity in the mutant EGFR NSCLC cells, with IC_50_ values of 12 nM, 6 nM and 2 nM against NCI-H1975, PC-9, and HCC827 cells, respectively (Table 1). However, it showed moderate or weak anti-growth activities in A431, LoVo and A549 cells, with IC_50_ values ranging from 338 nM to 1541 nM, which were 28- to 128-fold less potent than that in NCI-H1975 cells (Table 1).

**Table 1:** Effect of ASK120067 on cancer cells harboring mutant or wild-type EGFR

Cells	IC_50_(nM)
NCI-H1975	12 ± 4
PC-9	6 ± 3
HCC-827	2 ± 2
A431	388 ± 158
LoVo	1916 ± 1126
A549	1541 ± 359

ASK120067 inhibited the proliferation of cell lines harboring the T790M mutation (NCI-H1975 cells) or sensitizing mutations (PC-9 and HCC-827 cells), while it exhibited less potency against cell lines harboring EGFR ^*WT*^ (A431, LoVo and A549 cells). IC_50_ values are shown as the mean ± SD from three independent experiments

**Fig. 2:**
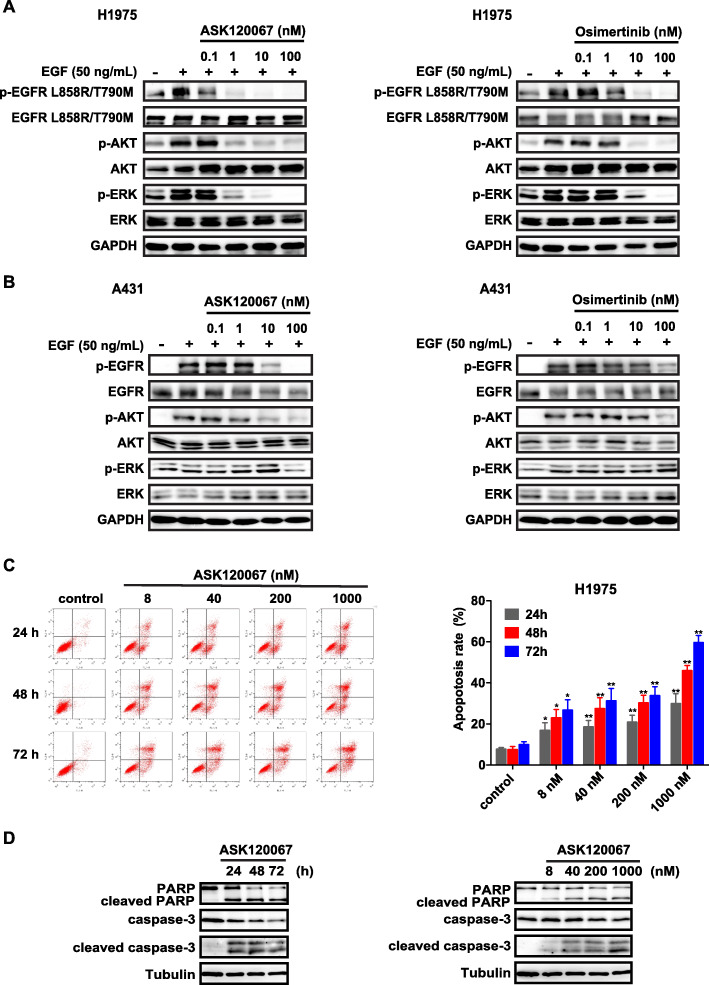
Effect of ASK120067 on cancer cells harboring mutant or wild-type EGFR. **a** and **b** ASK120067 or osimertinib inhibited the phosphorylation of EGFR at Tyrosine residue 1068 and its downstream signaling proteins AKT and ERK in NCI-H1975 cells (EGFR ^*L*858*R*/*T*790*M*^) **a** while showing less activity against the activation of EGFR and its downstream signaling in A431 cells expressing EGFR ^*WT*^**b**. **c** Apoptosis of NCI-H1975 cells was evaluated by flow cytometry after treatment with increasing concentrations of ASK120067 for 24 to 72 h. Data are plotted as the mean ± SEM, and significance of differences was determined by Student’s t test (^∗^*p* < 0.05, ^∗∗^*p* < 0.01). **d** The expression levels of cleaved PARP and cleaved caspase-3 in NCI-H1975 cells after ASK120067 treatment were determined by Western blot analysis

We then examined the effects of ASK120067 on EGFR signaling transduction in NCI-H1975, PC-9 and A431 cells by immunoblot analysis, and osimertinib treatment was used as positive control. ASK120067 dose-dependently inhibited EGF-induced EGFR ^*L*858*R*/*T*790*M*^ phosphorylation and consequent activation of the downstream molecules AKT and ERK in NCI-H1975 cells, with similar or even more effective potency than osimertinib (Fig. 2a). ASK120067 significantly inhibited p-EGFR ^*L*858*R*/*T*790*M*^ at a concentration as low as 0.1 nM and almost completely inhibited p-EGFR ^*L*858*R*/*T*790*M*^ at 1 to 10 nM (Fig. 2a). Similar results were obtained in PC-9 cells, as evidenced by the decreased levels of EGFR ^*exon*19*del*^ phosphorylation upon ASK120067 treatment (Fig. S1a). In contrast, the inhibitory activity of ASK120067 on EGFR ^*WT*^ phosphorylation in A431 cells was much weaker. As shown in Fig. 2b, quite similar to that with osimertinib treatment, the phosphorylation of EGFR ^*WT*^ was only partially inhibited by ASK120067 until the concentration reached 10 to 100 nM, demonstrating the selectivity of ASK120067 at the cellular level.

We further assessed the efficacy of ASK120067-induced apoptosis in NCI-H1975 and PC9 cells by Annexin-V/FITC staining and immunoblotting. ASK120067 treatment induced apoptosis in a dose- and time-dependent manner in both NCI-H1975 cells (Fig. 2c) and PC-9 cells (Fig. S1b), and the expression levels of cleaved PARP and caspase 3 proteins were increased upon ASK120067 treatment (Fig. 2d).

### ASK120067 demonstrated profound and selective antitumor efficacy in EGFR-mutant xenograft models in vivo

**Fig. 3:**
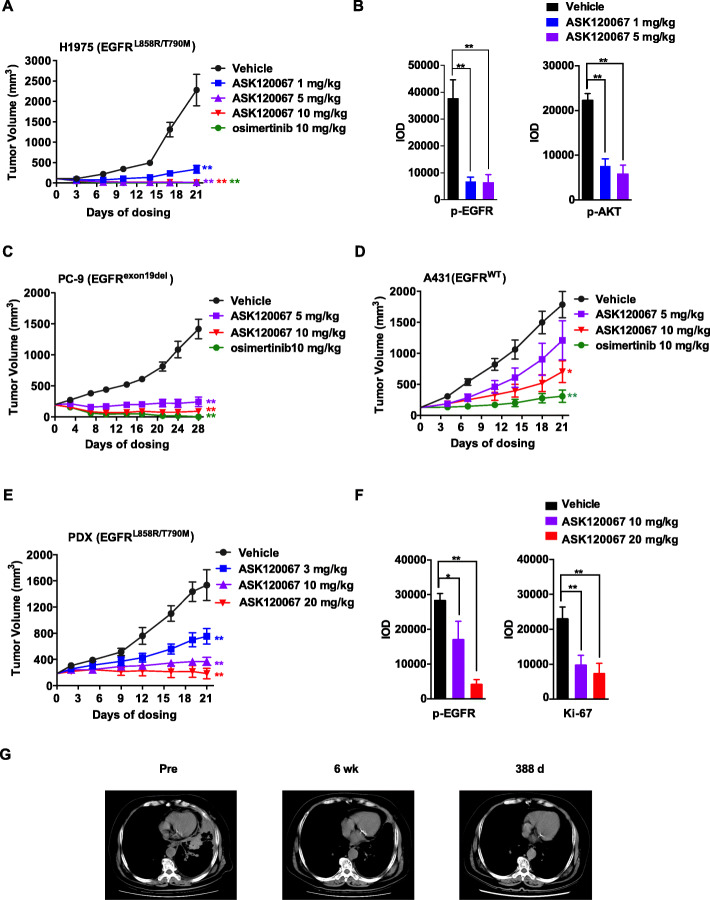
ASK120067 exerts in vivo antitumor activity against EGFR-mutant tumor xenograft models, and proof-of-concept clinical studies validate ASK120067 as an EGFR ^*T*790*M*^ inhibitor. **a** Antitumor activity of ASK120067 in the NCI-H1975 lung cancer xenograft model following 21 days of daily treatment with ASK120067 at doses of 1, 5, and 10 mg/kg/qd, with osimertinib (10 mg/kg/qd) as a positive control. **b** The phosphorylation of EGFR and AKT in tumor tissues of the NCI-H1975 xenograft model were evaluated by IHC staining after 21 days of treatment with ASK120067 or vehicle control and are presented by quantitative analysis. **c** Antitumor efficacy of ASK120067 in a PC-9 lung cancer xenograft model. **d** Antitumor activity of ASK120067 in the A431 epidermoid carcinoma xenograft model. **e** Antitumor efficacy of ASK120067 in PDX models harboring the EGFR ^*L*858*R*/*T*790*M*^ mutation. **f** The expression of phosphorylated EGFR and Ki-67 in PDX xenograft tumor tissues was evaluated by IHC staining and is presented by quantitative analysis. Data are presented as the mean ± SEM, and the significance of differences was determined by Student’s t test (^∗^*p* < 0.05, ^∗∗^*p* < 0.01). **g** Computed tomography scans of the chest from a patient before and after treatment with 40 mg ASK120067 in a phase I trial: images from a 74-year-old Chinese female diagnosed with stage IV EGFR-mutant (exon 19del) NSCLC in August 2016. The patient was previously treated with the first-line therapy icotinib for 16 months and achieved a partial response before eventually developing disease progression. See the main text for details

To explore the in vivo activity of ASK120067, we administered ASK120067 orally in model mice with NCI-H1975 xenografts at doses of 1, 5 or 10 mg/kg once daily; osimertinib was used as a positive control. As shown in Fig. 3a, ASK120067 dose-dependently inhibited tumor growth in the NCI-H1975 model. Administration of 1 mg/kg ASK120067 for 21 days resulted in significantly regressed tumor growth, with a tumor growth inhibition (TGI) rate of 85.7%, and administration of 10 mg/kg ASK120067 caused dramatic tumor shrinkage with a TGI rate of 99.3%, showing a similar potency with osimertinib (Fig. 3a). All doses of ASK120067 were well tolerated in animals, and no body weight loss was observed (data not shown). To validate the correlation between ASK120067 and antitumor activity, tumor tissues were analyzed with immunohistochemistry. As shown in the quantification data in Fig. 3b, ASK120067 significantly inhibited the phosphorylation of EGFR ^*L*858*R*/*T*790*M*^ and AKT in tumor tissue. Similar result was obtained in the PC-9 (EGFR^*exon19del*^) xenograft model, as demonstrated by significant tumor shrinkage after 28 days of ASK120067 treatment (5, 10 mg/kg once daily), with TGI rates of 86.0% and 93.0%, respectively (Fig. 3c). In contrast, ASK120067 administration induced less tumor regression in the A431 xenograft model (Fig. 3d). The TGI rate was less than 40% in mice treated with 5 mg/kg ASK120067, and the ratio only reached 61.8% at 10 mg/kg ASK120067, which is less effective than osimertinib (TGI rate of 82.9% at 10 mg/kg).

We further evaluated tumor responses in a patient-derived xenograft (PDX) model with lung tumor tissue harboring EGFR ^*L*858*R*/*T*790*M*^. ASK120067 treatment for 21 days induced increasing tumor regression at doses of 3 mg/kg, 10 mg/kg and 20 mg/kg with TGI rates of 56.8%, 76.7% and 85.2%, respectively (Fig. 3e). Immunohistochemistry analysis showed that ASK120067 significantly inhibited the phosphorylation of EGFR ^*L*858*R*/*T*790*M*^ in the tumor tissue of the PDX model after 21 days of treatment (Fig. 3f). Meanwhile, Ki-67 staining in tumor tissue was decreased after ASK120067 treatment (Fig. 3f), confirming the antiproliferative activity of ASK120067 in the EGFR ^*L*858*R*/*T*790*M*^ PDX model.

### ASK120067 caused EGFR ^*T*790*M*^ tumor shrinkage in a lung cancer patient with acquired resistance to early-generation EGFR TKIs in a clinical trial.

ASK120067 is currently in a phase I/II clinical trial (NCT03502850) comprising patients with advanced EGFR ^*T*790*M*^-positive NSCLC who had disease progression following treatment with EGFR inhibitors. Here, we present a preliminary result of a patient in this clinical study with a confirmed radiographic response after treatment with the lowest dose (40 mg once daily). The tumor from the patient contains drug-sensitive EGFR mutations in addition to documented T790M mutations (according to local testing results). The patient is a 74-year-old Chinese female diagnosed with stage IV EGFR-mutant (exon 19del) NSCLC in August 2016. The patient had a partial response to icotinib before undergoing disease progression after 16 months. Analysis of tumor tissue immediately before enrollment in the ASK120067 study revealed the presence of a T790M mutation (data not shown). A computed tomography scan of the chest of this patient before and after treatment with ASK120067 is shown in Fig. 3g. After treatment with ASK120067 for 6 weeks, the tumor in the lung almost completely disappeared, and the effect lasted more than one year as the patient continued to receive ASK120067 treatment. Further clinical studies of ASK120067 are ongoing in China.

### Acquired resistance to ASK120067 is associated with Ack1 activation in T790M-positive NCI-H1975 cells

To further address potential acquired resistance to ASK120067, we continuously exposed NCI-H1975 cells to increasing doses of this compound or osimertinib for approximately 4 months until resistance developed. There was a greater than 100-fold change in the IC_50_ values of ASK120067-resistant cell populations (67R) or osimertinib-resistant cell populations (AZDR) compared to parental cells (Fig. S2a), and we also observed cross-resistance between this two compounds (Fig. S2b). Whole genome sequencing (WGS) revealed no additional mutations in EGFR or any of the other oncogenes tested in resistant cells (data not shown). However, the original L858R/T790M mutation was present but at lower levels in 67R and AZDR (Supplementary Fig. 2c).

**Fig. 4:**
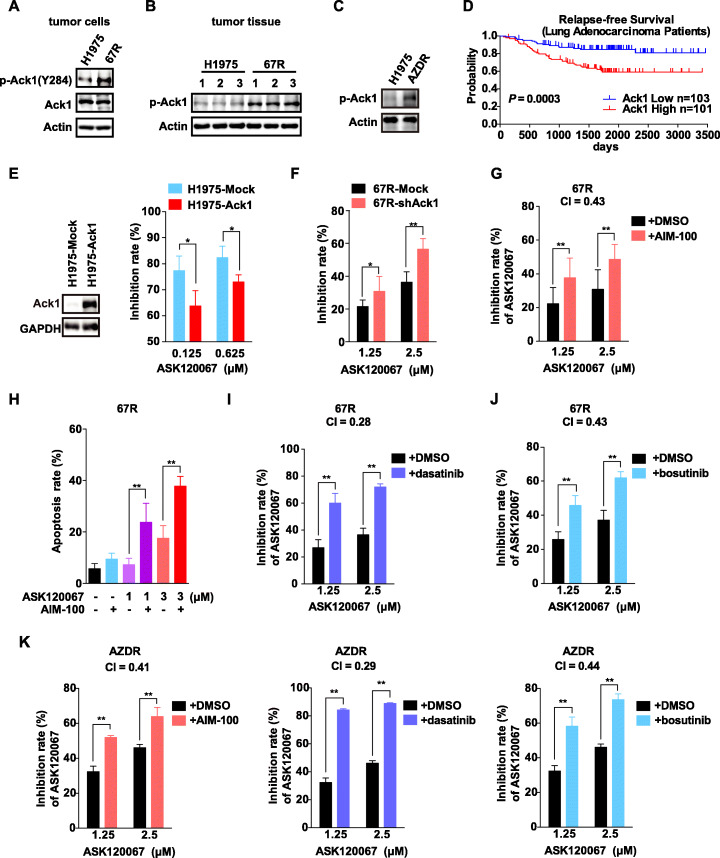
Activation of Ack1 is sufficient to cause resistance to ASK120067, and drug combinations suppress proliferation and induce apoptosis of ASK120067-resistant cells. **a** Immunoblot analysis of total Ack1 and phosphorylated Ack1 (p-Ack1) levels in parental NCI-H1975 and 67R cells. **b** The expression of p-Ack1 in NCI-H1975 xenograft tumors and 67R xenograft tumors was compared by immunoblotting. **c** The p-Ack1 protein in AZDR and NCI-H1975 was detected by immunoblotting. **d** Correlation analysis of Ack1 expression and relapse-free survival (RFS) of 204 lung adenocarcinoma cancer patients (GSE22138) is presented as a Kaplan-Meier plot. **e** The antiproliferation effects of ASK120067 on NCI-H1975 cells ectopically expressing negative control vector or Ack1 were assessed using an SRB assay. **f** Knockdown of Ack1 expression in 67R effectively enhanced the antiproliferation potency of ASK120067. **g** ASK120067 in combination with AIM-100 caused a significantly higher growth-inhibition rate in ASK120067-resistant cells than that with ASK120067 treatment alone. **h** AIM-100 synergistically enhanced the apoptosis-inducing activity of ASK120067 in 67R cells. Cells were treated with ASK120067, AIM-100 alone or both drugs in combination for 48 h, and apoptosis was assessed using flow cytometry. **i** and **j** Combination ASK120067 with dasatinib **i** or bosutinib **j** partially restored the growth-inhibition sensitivity of ASK120067-resistant cells to ASK120067 treatment. **k** Combination ASK120067 with AIM-100, dasatinib or bosutinib synergistically inhibited the growth of AZDR cells. Data are plotted as the mean ± SD, and significance of differences was evaluated by Student’s *t* test (^∗^*p* < 0.05, ^∗∗^*p* < 0.01)

As bypass signaling is a common form of resistance to kinase inhibitors, we sought to identify potential kinases that are activated in ASK120067-resistant cells. We performed a human phospho-RTK array and compared the differential expression of 71 tyrosine kinases in parental and resistant NCI-H1975 cells. We found that the phosphorylation of Ack1 was dramatically upregulated in resistant cells (Supplementary Fig. 3a). We further confirmed the upregulated Ack1 phosphorylation (p-Ack1) in the 67R cells by Western blotting analysis, and no increase in total Ack1 protein was observed (Fig. 4a). To extend the in vitro observations concerning the upregulation of p-Ack1 in resistant cells, we subcutaneously injected NCI-H1975 parental or 67R cells into athymic mice. In line with the in vitro results, the expression of p-Ack1 in ASK120067-resistant tumors was significantly higher than that in NCI-H1975 tumors (Fig. 4b). Moreover, we also examined the Ack1 activation in osimertinib-resistant cells and Ack1 was found to be highly activated in AZDR cells compared with the parental cells (Fig. 4c). Furthermore, to identify any possible relationship between Ack1 and NSCLC, a Kaplan-Meier plotter analysis was performed, and we found that the expression level of Ack1 was inversely correlated with survival time in NSCLC patients (*p* < 0.01) (Fig. 4d). Based on these data, we speculated that Ack1 might be a key protein mediating resistance to the third-generation EGFR inhibitors.

### Ack1 drives resistance to ASK120067 through the AKT-BIM pathway

As hyperactivation of Ack1 was detected in ASK120067-resistant cells and tumors and was associated with the survival of NSCLC patients, its role in resistance was further investigated. We first asked whether overexpression of Ack1 is sufficient to confer resistance to ASK120067. The results showed that ectopic expression of Ack1 rendered NCI-H1975 cells less sensitive to ASK120067 (Fig. 4e). In contrast, depletion of Ack1 in 67R cells using specific shRNA not only significantly decreased cell proliferation (Fig. S3b) but also restored partial sensitivity of resistant cells to ASK120067 (Fig. 4f). We next sought to determine whether this finding might suggest effective combinations of available therapies. AIM-100, a highly selective inhibitor of Ack1, was used [27]. Combining ASK120067 with AIM-100 produced synergistic antiproliferative efficacy in 67R (Fig. 4g), with combination index (CI) value of 0.43. As we noticed that 67R cells showed apoptosis resistance to ASK120067 treatment (Fig. S4), we speculated that apoptosis resistance was involved in the resistance of 67R cells and thus pursued whether the Ack1 inhibitor could restore the apoptosis-inducing activity of ASK120067. The data showed that concomitant treatment of ASK120067 with AIM-100 largely enhanced apoptosis of 67R cells compared with ASK120067 treatment alone (Fig. 4h). Furthermore, two other clinically relevant TKIs with potent anti-Ack1 activity, dasatinib and bosutinib [27], were used in combination with ASK120067. Both dasatinib and bosutinib with ASK120067 effectively improved cell growth inhibition (Fig. 4i and 4j) and increased apoptosis compared with ASK120067 alone (Fig. S5). Furthermore, the efficacy of the above combination strategies was verified in the osimertinib-resistant AZDR cells, and similar effects were observed with each CI value less than 0.5 (Fig. 4k).

**Fig. 5:**
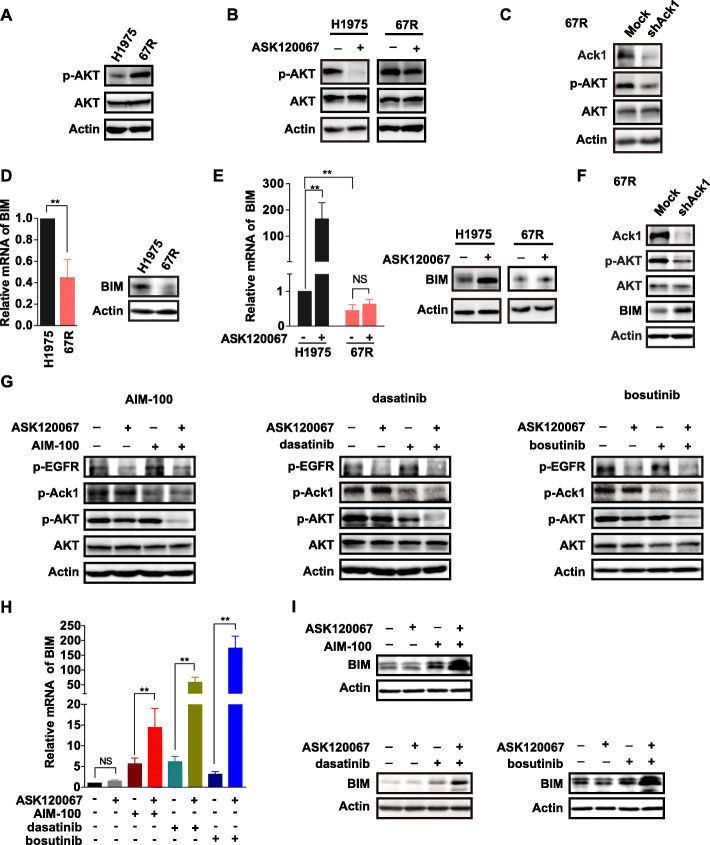
Activation of antiapoptotic signaling through the Ack1/AKT pathway contributes to ASK120067 resistance. **a** The levels of AKT phosphorylation (p-AKT) in NCI-H1975 and 67R cells were determined by immunoblotting analysis. **b** The inhibitory activity of ASK120067 on p-AKT expression in NCI-H1975 cells and 67R cells was compared. **c** Knockdown of Ack1 expression using short hairpin RNA (shRNA) decreased the levels of phosphorylated AKT in 67R cells. **d** The mRNA and protein levels of proapoptotic protein BIM in NCI-H1975 and 67R cells were determined by real-time PCR (left panel) and Western blot analysis (right panel), respectively. **e** The effect of ASK120067 on BIM expression in NCI-H1975 and 67R cells was examined. **f** Knockdown of Ack1 expression in 67R cells increased the expression of BIM by decreasing the phosphorylation of AKT. **g** to **i**, the combination of ASK120067 with Ack1 inhibitors synergistically suppressed AKT activation **g** and induced the transcription **h** and protein expression of BIM **i**

To understand how Ack1 might regulate apoptosis and proliferation, we probed several signaling pathways known to be associated with resistance to EGFR TKIs. High basal levels of phosphorylated AKT (p-AKT) were observed in 67R cells compared with the parental cell line (Fig. 5a). Meanwhile, ASK120067 resulted in the complete reduction of p-AKT to negligible levels in the NCI-H1975 parental cells; in contrast, it had no obvious inhibition of p-AKT in resistant cells (Fig. 5b). Moreover, if Ack1 was knocked down in 67R cells, the phosphorylation of AKT was decreased accordingly (Fig. 5c). These data suggest that hyperphosphorylation of Ack1 in resistant cells leads to AKT activation and thus decreases the antitumor effects of ASK120067.

To determine how AKT engages the apoptotic machinery, we examined the expression of the downstream proapoptotic factor Bcl-2-like protein 11 (BIM) because its induction is essential for cell death induced by EGFR TKIs [41, 42]. The data showed that the expression of BIM was downregulated at the mRNA and protein levels in 67R cells compared with the NCI-H1975 parental cells (Fig. 5d). ASK120067 treatment alone failed to increase the expression of BIM protein in 67R cells, although it potently upregulated BIM expression in parental cells (Fig. 5e). Moreover, the expression of BIM was increased in 67R cells when Ack1 expression was knocked down and AKT activation was subsequently downregulated (Fig. 5f), indicating that BIM protein is involved in ASK120067 resistance driven by the Ack1-AKT pathway.

We subsequently examined the influence of therapeutic combinations of ASK120067 and Ack1 inhibitors on target inhibition, AKT activation and BIM expression in resistant cells. We found that the addition of either ASK120067 or each Ack1 inhibitor alone, including AIM-100, dasatinib or bosutinib, to ASK120067-resistant cells resulted in obviously decreased phosphorylation of EGFR or Ack1 and a weak to moderate decrease of AKT phosphorylation (Fig. 5g). Intriguingly, the combination of ASK120067 with any one of the Ack1 inhibitors led to complete inhibition of AKT phosphorylation (Fig. 5g). Meanwhile, the combination of ASK120067 with the Ack1 inhibitors synergistically upregulated BIM expression at both the mRNA (Fig. 5h) and protein levels (Fig. 5i) in 67R cells. These results confirmed that targeting both EGFR and Ack1 could effectively induce apoptosis of ASK120067-resistant cells through inhibition of the AKT-BIM pathway.

### ASK120067 and Ack1 inhibitors have a synergistic effect in ASK120067-resistant cells in vivo

**Fig. 6:**
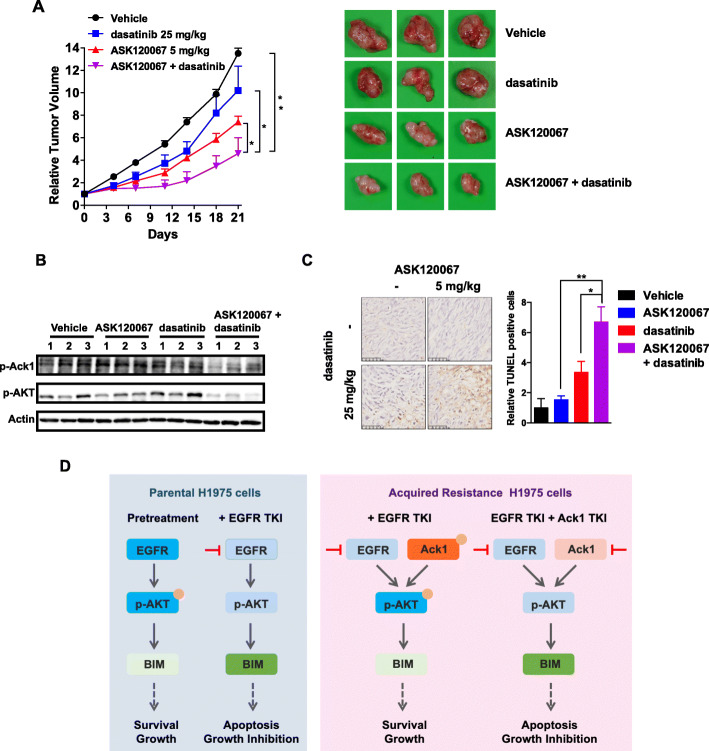
Combination therapy with ASK120067 and Ack1 inhibitors showed synergistic in vivo antitumor effects in the 67R xenograft model. **a** Growth of 67R xenograft tumors following daily treatment with 5 mg/kg ASK120067, 25 mg/kg dasatinib or a combination of ASK120067 and dasatinib for 21 days. **b** The expression levels of p-Ack1 and p-AKT in 67R xenograft tumors were assessed after 21 days of treatment. **c** Cell apoptosis in 67R xenograft tumors was tested by the TUNEL assay after 21 days of treatment. Significance of differences was determined by Student’s t test (^∗^*p* < 0.05, ^∗∗^*p* < 0.01). **d** Proposed mechanism for the efficacy of the combination strategy in ASK120067-resistant NCI-H1975 cells

Finally, we tested the concept that Ack1 inhibition could circumvent resistance to ASK120067 using in vivo mouse models with tumors with ASK120067 resistance. ASK120067 and dasatinib were orally administered once daily at doses of 5 mg/kg and 25 mg/kg, respectively. We observed that ASK120067 exhibited less potency against the growth of ASK120067-resistant tumors than of NCI-H1975 tumors, indicating the maintenance of drug resistance of ASK120067-resistant cells in vivo (Fig. S6). Meanwhile, dasatinib treatment alone exhibited no obvious effect on tumor growth (Fig. 6a). In contrast, tumor growth was significantly suppressed in the combination therapy group compared to the control (*p* < 0.01), ASK120067 alone (*p* < 0.05), and dasatinib monotherapy groups (*p* < 0.05) (Fig. 6a). Following combination treatment, a more profound effect on AKT phosphorylation levels was observed than that in response to either agent alone (Fig. 6b), together with an increase in apoptotic cells in tumors based on TUNEL results (Fig. 6c). Collectively, the results confirmed that the combination of EGFR inhibitor and Ack1 inhibitor induces apoptosis and acts synergistically to suppress the growth of cells with acquired resistance in vivo.

On the basis of our findings, the potential mechanism whereby Ack1/AKT signaling attenuates the effects of ASK120067 in EGFR-mutant cells is shown schematically in Fig. 6d. Continuous inhibition of EGFR signaling by ASK120067 can activate Ack1, leading to sustained AKT phosphorylation and subsequent apoptosis resistance. Ack1 inhibitors enhance the effects of ASK120067 by directly inhibiting Ack1/AKT signaling.

## Discussion

NSCLC accounts for 85% of malignant lung tumors and is the leading cause of cancer-related death [43]. Although EGFR TKIs are clinically effective in NSCLC patients who have EGFR oncogene mutations, the EGFR T790M acquired mutation is still a major clinical challenge for NSCLC therapy [44, 45]. Here, we describe the identification and early clinical development of ASK120067, a novel third-generation EGFR TKI. Kinase profiling together with cellular studies have shown that ASK120067 is highly potent against EGFR T790M-resistant and EGFR-sensitizing mutants, with good selectivity against EGFR ^*WT*^. Meanwhile, the in vivo antitumor activity of ASK120067 across xenograft and PDX models suggests the potential to target T790M tumors following acquired resistance to early-generation TKIs. Moreover, the confirmed response described in this article demonstrated proof-of-principle clinical activity in a patient with T790M acquired resistance. ASK120067 is currently in phase I/II clinical trials to evaluate its safety, pharmacokinetics, and preliminary efficacy in previously treated patients with EGFR-mutant NSCLC. Evidence of efficacy has been observed at all doses studied so far, and ASK120067 seems to be well tolerated with no evidence of serious side effects. In addition, in vitro results suggested the potential of ASK120067 to target other kinase that possess a conserved cysteine equivalent to C797 in EGFR, including HER2 and ITK [24]. As *HER2* amplification was reported to be involved in acquired resistance to EGFR TKI, and meanwhile, ITK has been shown to be aberrantly activated in T-cell malignancies, further studies are warranted to evaluate the activity of ASK120067 against these kinases in lung cancer and in T-cell malignancies, respectively.

Understanding the mechanisms underlying resistance to EGFR TKIs is important because it allows treatment strategies to be adjusted and provides patients with the best possible clinical care. We thus investigated the potential resistance mechanism of ASK120067 in this study and determined the effective rational drug combination to override the acquired resistance to ASK120067. Hyperactivation of Ack1 was identified as an important contributor to both ASK120067 resistance and osimertinib resistance, indicating that Ack1 may act as a common drug-resistance marker of the third generation EGFR inhibitors. To the best of our knowledge, this is the first study to reveal that the activation of Ack1 is involved in the resistance of targeted anticancer drugs. Mechanistic investigation revealed that hyperphosphorylation of Ack1 and subsequent activation of antiapoptotic signaling via the AKT pathway contributes to ASK120067 resistance. In previous studies, AKT pathway reactivation was reported to play an important role in cancer cell survival in response to EGFR TKI treatment. Researchers have demonstrated that SFK and focal adhesion kinase (FAK) sustained AKT and MAPK signaling under continuous EGFR inhibition in osimertinib-sensitive cells. In addition, amplification of insulin-like growth factor 1 receptor (IGF1R) expression was involved in the overactivation of AKT and contributed greatly to resistance against third-generation EGFR TKIs. Our research provided further evidence that hyperphosphorylation of Ack1 might account for, at least in part, the activation of AKT in EGFR TKI-resistant cells. Meanwhile, it also supports an important notion that Ack1 is a potential druggable target in cells resistant to third-generation EGFR TKIs.

Very few studies have been conducted in the development of Ack1 inhibitors. Dasatinib, bosutinib and AIM-100 have been previously reported as Ack1 inhibitors [31]. Among them, dasatinib and bosutinib are multikinase inhibitors with targets that include Bcr/Abl, Src and Ack1, while AIM-100 is a specific and the best-studied Ack1 inhibitor. Despite the ability of AIM-100 to inhibit cancer cell proliferation, this compound has not progressed further as a prospective therapeutic agent because of its limited solubility in aqueous environments [27]. As Ack1 inhibitors have been proven to be of crucial importance to counter the resistance of hormone-dependent tumors, and, importantly, our data showed for the first time that blocking Ack1 could be a new therapeutic approach to overcome drug resistance of EGFR TKI in lung cancer, it is worthwhile to develop novel and selective Ack1 inhibitors in the near future.

Given the observation that multiple distinct mechanisms of drug resistance can develop simultaneously within the same patient at the time of relapse, Ack1 activation might also coexist with other factors driving resistance. We noticed that the combination of ASK120067 with dasatinib was more efficacious than that of ASK120067 and AIM-100 at increasing apoptosis and inhibiting cell proliferation resistant cells, although there was no difference at inhibiting AKT phosphorylation. These data suggest that there are additional factors that attenuate the effects of ASK120067 through AKT or other signaling pathways and that these factors can be inhibited by dasatinib. Notably, the combination of a first-generation EGFR TKI plus dasatinib was evaluated in a phase II clinical trial in patients with EGFR-mutant lung cancer and acquired resistance to either gefitinib or erlotinib [46]. Moreover, a phase I/II clinical trial of osimertinib plus dasatinib in EGFR-TKI-naïve patients recently began in the United States (NCT02954523). These combination therapies are mainly based on the result that Src activation contributes to the resistance to EGFR-TKIs in EGFR-mutant NSCLC, and the combination of an EGFR-TKI and a Src inhibitor is synergistic in these tumors. Our findings that Ack1 plays a critical role in EGFR TKI resistance and that combining dasatinib with ASK120067 effectively restored the sensitivity of ASK120067 in resistant tumors provide a new set of mechanisms to support the clinical combination of EGFR TKIs with dasatinib for the treatment of EGFR-mutant NSCLC.

## Conclusions

We identified ASK120067 as a novel third-generation EGFR TKI in this work and provide mechanistic insights into the bypass signaling in the setting of drug resistance to the third-generation EGFR TKIs, which supports the use of rational combination therapies for clinical use. Our findings should prompt further investigations of third-generation EGFR inhibitors and Ack1 inhibitors as a combination therapy in patients whose tumors harbor EGFR mutations.

## Supplementary information

**Additional file 1** Figure S1. ASK120067 treatment inhibits activation of EGFR and downstream signaling and induces apoptosis in PC-9 cells. Figure S2. characterization of ASK120067- or osimertinib resistant NCI-H1975 cell lines. Figure S3. ASK120067-resistant NCI-H1975 cells exhibited high Ack1 phosphorylation levels and growth dependence on Ack1. Figure S4. ASK120067-resistant cells exhibited apoptotic resistance to ASK120067 treatment. Figure S5. combination of ASK120067 with either dasatinib **a** or bosutinib **b** partially restored the apoptosis-inducing activity of ASK120067-resistant cells to ASK120067 treatment. Figure S6. comparison of the in vivo antitumor efficacy of ASK120067 in an NCI-H1975 xenograft model and ASK120067-resistant xenograft models.

**Additional file 2** Supplementary materials and methods.

## Data Availability

All data in this study are available upon reasonable request.

## Abbreviations

NSCLC: non-small cell lung cancer
EGFR ^*W**T*^: wild-type EGFR
TKI: tyrosine kinase inhibitor
RTK: receptor tyrosine kinase
EMT: epithelial-to-mesenchymal transition
FDA: U.S. Food and Drug Administration
ELISA: enzyme-linked immunosorbent assay
IHC: immunohistonchemistry
PDX: patient-derived xenograft
TGI: tumor growth inhibition
WGS: whole genome sequencing
qRT-PCR: quantitative real-time PCR
CI: combination index
TUNEL: terminal deoxynucleotidyl transferase (TdT)-mediated dUTP nick end labelin
