# Correction to: Discovery of a novel third-generation EGFR inhibitor and identification of a potential combination strategy to overcome resistance

**DOI:** 10.1186/s12943-021-01391-x

**Published:** 2021-08-17

**Authors:** Tao Zhang, Rong Qu, Shingpan Chan, Mengzhen Lai, Linjiang Tong, Fang Feng, Hongyu Chen, Tingting Song, Peiran Song, Gang Bai, Yingqiang Liu, Yanan Wang, Yan Li, Yi Su, Yanyan Shen, Yiming Sun, Yi Chen, Meiyu Geng, Ke Ding, Jian Ding, Hua Xie

**Affiliations:** 1 Division of Antitumor Pharmacology, State Key Laboratory of Drug Research, Shanghai Institute of Materia Medica, Chinese Academy of Sciences, 555 Zuchongzhi Road, Shanghai, 201203 China; 2 International Cooperative Laboratory of Traditional Chinese Medicine Modernization and Innovative Drug Development of Chinese Ministry of Education (MOE), Guangzhou City Key Laboratory of Precision Chemistry Drug Development, School of Pharmacy, Jinan University, No. 601 Huangpu Avenue West, Guangzhou, 510632 China; 3 School of Pharmacy, Fudan University, 826 Zhangheng Road, Shanghai, 201203 China; 4 Jiangsu Aosaikang Pharmaceutical Co.Ltd (ASK pharm), 699 Kejian Road, Nanjing, 211112 China; 5 University of Chinese Academy of Sciences, 19A Yuquan Road, Beijing, 100049 China; 6 School of Life Science and Technology, ShanghaiTech University, 393 Middle Huaxia Road, Shanghai, 201210 China

**Correction to: Mol Cancer 19, 90 (2020)**

**https://doi.org/10.1186/s12943-020-01202-9**

Following the publication of the original paper [1], the authors found minor typographical errors that should be corrected.

In the Results section, the dosage of ASK120067 in the sentence “we present a preliminary result of a patient in this clinical study with a confirmed radiographic response after treatment with the lowest dose (**40 mg/kg** once daily)” has been corrected to “…with the lowest dose (**40 mg** once daily)”.

In the figure legend of Figure 3g, the sentence “Computed tomography scans of the chest from a patient before and after treatment with **40 mg/kg** ASK120067 in a phase I trial…” has been corrected to “…treatment with **40 mg** ASK120067 once daily in a phase I trial…”.

These corrections have no impact on the results or conclusions of the study.

The original article has been corrected.
