# Correction: Discovery of a novel third-generation EGFR inhibitor and identification of a potential combination strategy to overcome resistance

**DOI:** 10.1186/s12943-023-01842-7

**Published:** 2023-08-19

**Authors:** Tao Zhang, Rong Qu, Shingpan Chan, Mengzhen Lai, Linjiang Tong, Fang Feng, Hongyu Chen, Tingting Song, Peiran Song, Gang Bai, Yingqiang Liu, Yanan Wang, Yan Li, Yi Su, Yanyan Shen, Yiming Sun, Yi Chen, Meiyu Geng, Ke Ding, Jian Ding, Hua Xie

**Affiliations:** 1 Division of Antitumor Pharmacology, State Key Laboratory of Drug Research, Shanghai Institute of Materia Medica, Chinese Academy of Sciences, 555 Zuchongzhi Road, Shanghai, 201203 China; 2 International Cooperative Laboratory of Traditional Chinese Medicine Modernization and Innovative Drug Development of Chinese Ministry of Education (MOE), Guangzhou City Key Laboratory of Precision Chemistry Drug Development, School of Pharmacy, Jinan University, No. 601 Huangpu Avenue West, Guangzhou, 510632 China; 3 School of Pharmacy, Fudan University, 826 Zhangheng Road, Shanghai, 201203 China; 4 Jiangsu Aosaikang Pharmaceutical Co.Ltd (ASK Pharm), 699 Kejian Road, Nanjing, 211112 China; 5 University of Chinese Academy of Sciences, 19A Yuquan Road, Beijing, 100049 China; 6 School of Life Science and Technology, ShanghaiTech University, 393 Middle Huaxia Road, Shanghai, 201210 China

**Correction: Mol Cancer 19, 90 (2020)**

**https://doi.org/10.1186/s12943-020-01202-9**

Following publication of the original article [1], the authors do notice that they mis-claimed the qRT-PCR primers that predicted to target human CDH10 gene as specific primers targeting human BIM gene in Materials and methods section when describing quantitative RT-PCR, and the primers have been mis-used to detect mRNA levels of BIM in Fig. 5d, e and h. They are extremely sorry for the misleading caused by this mistake, and to correctly quantify BIM mRNA levels in their tumor cell models, they have re-designed new specific qRT-PCR primers targeting human BIM (forward primer, 5’-TAAGTTCTGAGTGTGACCGAGA-3’, reverse primer, 5’-GCTCTGTCTGTAGGGAGGTAGG-3’), and repeated the qRT-PCR experiments in Fig. 5d, e and h. As shown below, they observed the similar results as our previous data, which was also consistent with the protein levels of Bim previously determined by immunoblotting in Fig. 5d, e and i. They believe that this mistake could be correct and would not affect their critical conclusions in our published paper. The corrected Fig. 5 and updated data of corrected RT-PCR results are provided below.

**Fig. 5:**
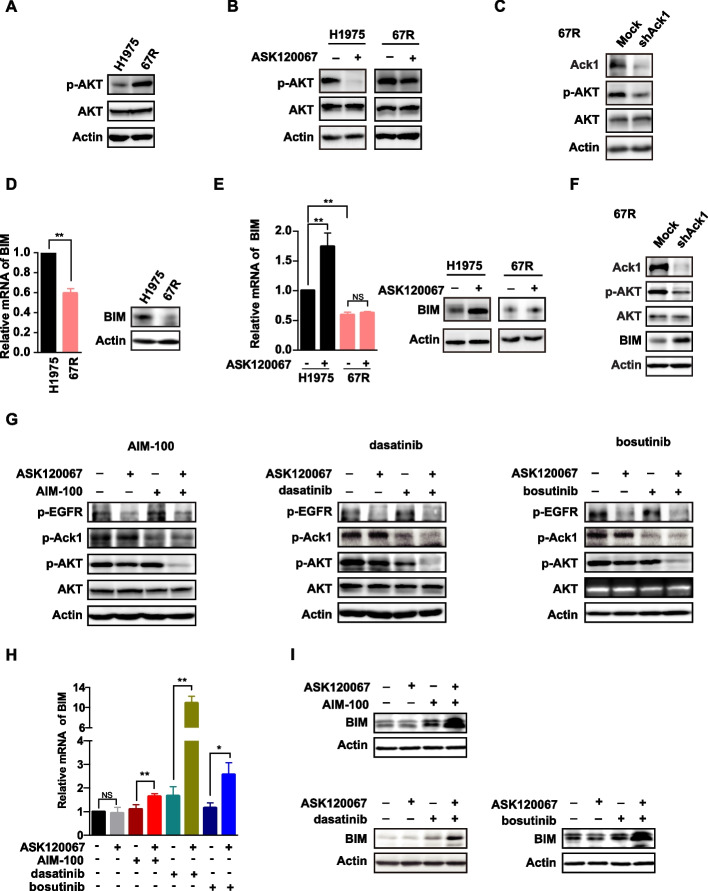
Activation of antiapoptotic signaling through the Ack1/AKT pathway contributes to ASK120067 resistance. **a** The levels of AKT phosphorylation (p-AKT) in NCI-H1975 and 67R cells were determined by immunoblotting analysis. **b** The inhibitory activity of ASK120067 on p-AKT expression in NCIH1975 cells and 67R cells was compared. **c** Knockdown of Ack1 expression using short hairpin RNA (shRNA) decreased the levels of phosphorylated AKT in 67R cells. **d** The mRNA and protein levels of proapoptotic protein BIM in NCI-H1975 and 67R cells were determined by real-time PCR (left panel) and Western blot analysis (right panel), respectively. **e** The effect of ASK120067 on BIM expression in NCI-H1975 and 67R cells was examined. **f** Knockdown of Ack1 expression in 67R cells increased the expression of BIM by decreasing the phosphorylation of AKT. **g** to **i**, the combination of ASK120067 with Ack1 inhibitors synergistically suppressed AKT activation **g** and induced the transcription **h** and protein expression of BIM **i**

**Quantitative RT-PCR**

Cells were treated with DMSO or the indicated compounds for 48 h before being subjected to RNA purification via an EZ-press Cell to cDNA Kit (EZBioscience, #B0001). Samples were then analyzed for mRNA expression via qRT-PCR using the iTaq TM Universal SYBR Ⓡ Green Supermix (BioRad, #1725125) and 7500 real-time PCR instrument (Applied Biosystems). The primer sequences were as follows: BIM, forward primer, 5’-TAAGTTCTGAGTGTGACCGAGA-3’, reverse primer, 5’-GCTCTGTCTGTAGGGAGGTAGG-3’; ACTIN, forward primer, 5’-CACCATTGGCAATGAGCGGTTC-3’, reverse primer, 5’-AGGTCTTTGCGGATGTCCACGT-3’. Primer synthesis was completed by TsingkeBiotechnology.
