# Supplementary material for: Discovery of a novel third-generation EGFR inhibitor and identification of a potential combination strategy to overcome resistance
Source: Mol Cancer. 2020 May 13;19:90. doi: 10.1186/s12943-020-01202-9 (PMC7218543; doi:10.1186/s12943-020-01202-9)
Supplement: Supplementary file 1 — Additional file 1 Figure S1. ASK120067 treatment inhibits activation of EGFR and downstream signaling and induces apoptosis in PC-9 cells. Figure S2. characterization of ASK120067- or osimertinib resistant NCI-H1975 cell lines. Figure S3. ASK120067-resistant NCI-H1975 cells exhibited high Ack1 phosphorylation levels and growth dependence on Ack1. Figure S4. ASK120067-resistant cells exhibited apoptotic resistance to ASK120067 treatment. Figure S5. combination of ASK120067 with either dasatinib a or bosutinib b partially restored the apoptosis-inducing activity of ASK120067-resistant cells to ASK120067 treatment. Figure S6. comparison of the in vivo antitumor efficacy of ASK120067 in an NCI-H1975 xenograft model and ASK120067-resistant xenograft models. [file 12943_2020_1202_MOESM1_ESM.pdf]

Supplementary Figure 1

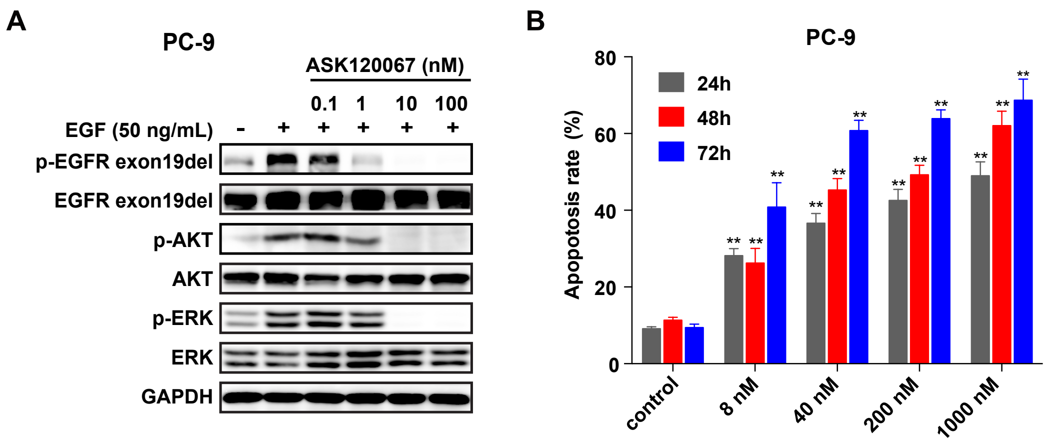

Fig. S1. ASK120067 treatment inhibits activation of EGFR and downstream signaling and induces apoptosis in PC-9 cells. **a** ASK120067 inhibited the phosphorylation of EGFR-Tyr1068 and its downstream signaling proteins AKT and ERK in PC-9 cells. **b** Apoptosis rate of PC-9 cells was evaluated by flow cytometry after treatment with increasing concentrations of ASK120067 for 24 to 72 h. Data are presented as the mean ± SEM, and the significance of differences was determined by Student's *t* test (**p* < 0.05, ***p* < 0.01).

Supplementary Figure 2

A

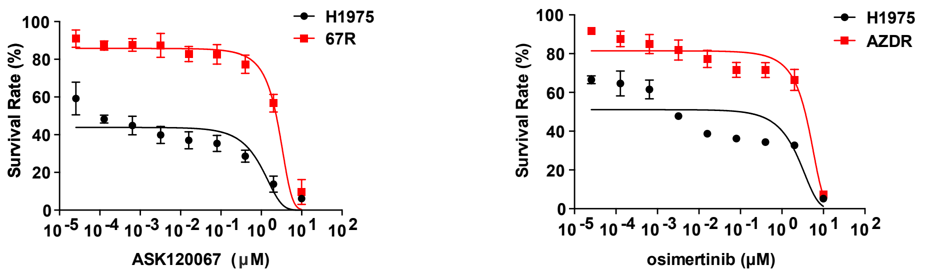

B

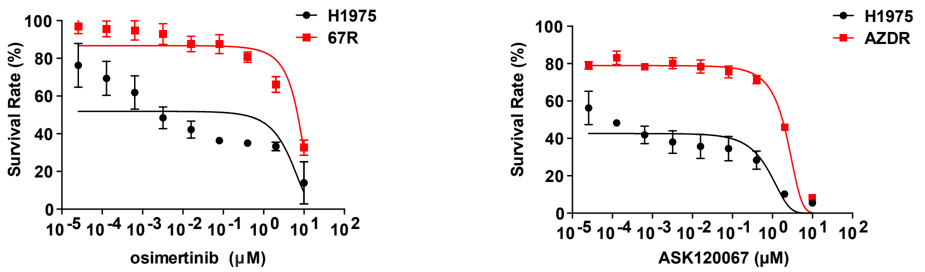

C

Cells	EGFR codon 790		EGFR codon 858	
	Numbers of reads indicate T	Numbers of reads indicate M	Numbers of reads indicate L	Numbers of reads indicate R
H1975	12	52 (81.25%)	25	66 (72.53%)
67R	24	51 (68.00%)	33	57 (63.33%)
AZDR	31	65 (67.71%)	42	80 (65.57%)

Fig. S2 Characterization of ASK120067- or osimertinib resistant NCI-H1975 cell lines. **a** ASK120067-resistant cell populations (67R) or osimertinib-resistant cell populations (AZDR) and the parental NCI-H1975 cells were treated with the indicated concentrations of ASK120067 or osimertinib for 72 h. **b** 67R or AZDR and the parental NCI-H1975 cells were treated with the indicated concentrations of osimertinib or ASK120067 for 72 h. Viable cells were measured by the SRB assay and plotted relative to the untreated controls. **c** Mutation status of the 790 codon and 858 codon of EGFR in NCI-H1975, 67R and AZDR cells were tested by whole-exome sequencing (WES).

Supplementary Figure 3

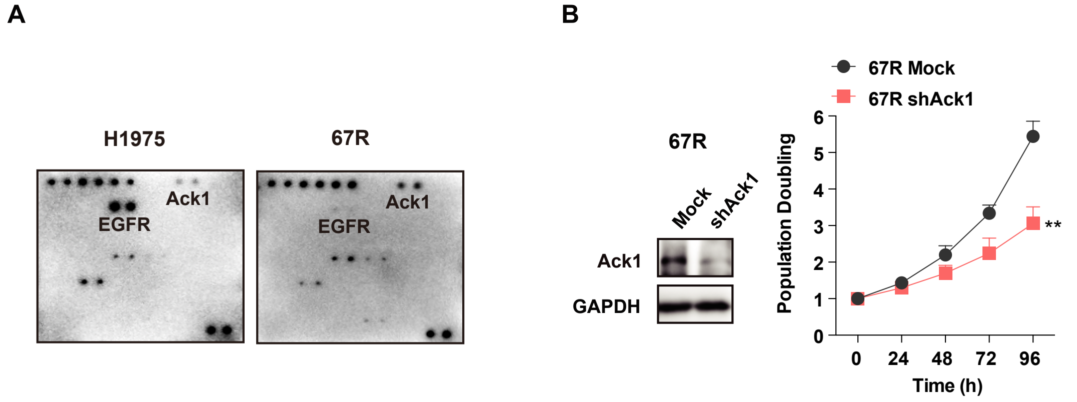

Fig. S3 ASK120067-resistant NCI-H1975 cells exhibited high Ack1 phosphorylation levels and growth dependence on Ack1. **a** Phosphorylation levels of 71 kinases in parental NCI-H1975 cells and 67R cells were tested and compared using human tyrosine kinase phosphorylation array. **b** Growth curves of ASK120067-resistant NCI-H1975 cells after Ack1 knockdown were assessed by SRB assay. Data are shown as the mean ± SEM, and the significance of differences was calculated by Student's *t* test (*$p < 0.05$, **$p < 0.01$).

Supplementary Figure 4

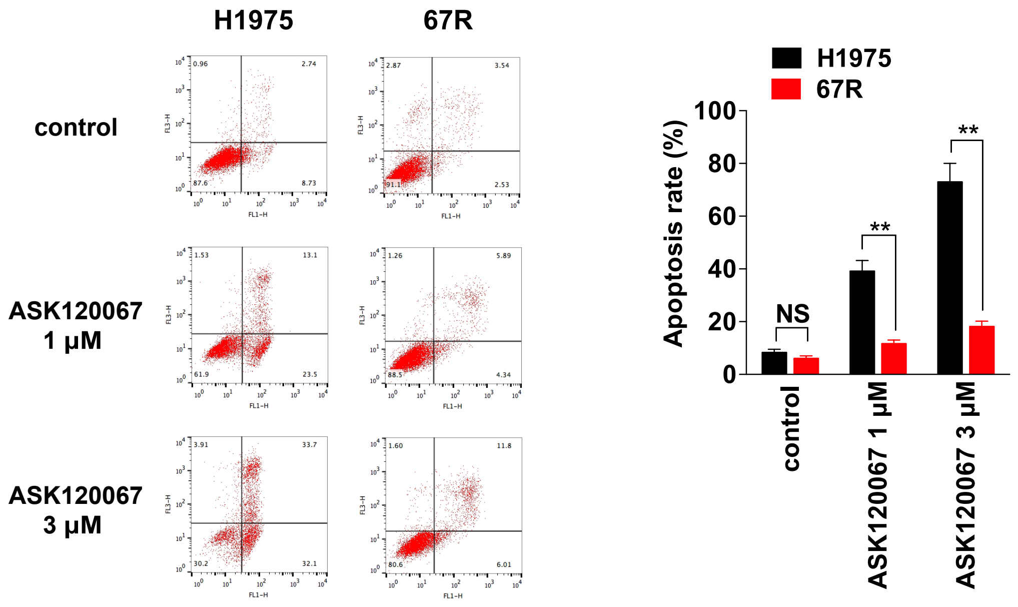

Fig. S4 ASK120067-resistant cells exhibited apoptotic resistance to ASK120067 treatment. 67R and the parental NCI-H1975 cells were treated with ASK120067 for 48 h, and then apoptosis rates were evaluated by flow cytometry. Data are presented as the mean ± SEM, and the significance of differences was determined by Student's *t* test (*$p < 0.05$, **$p < 0.01$).

Supplementary Figure 5

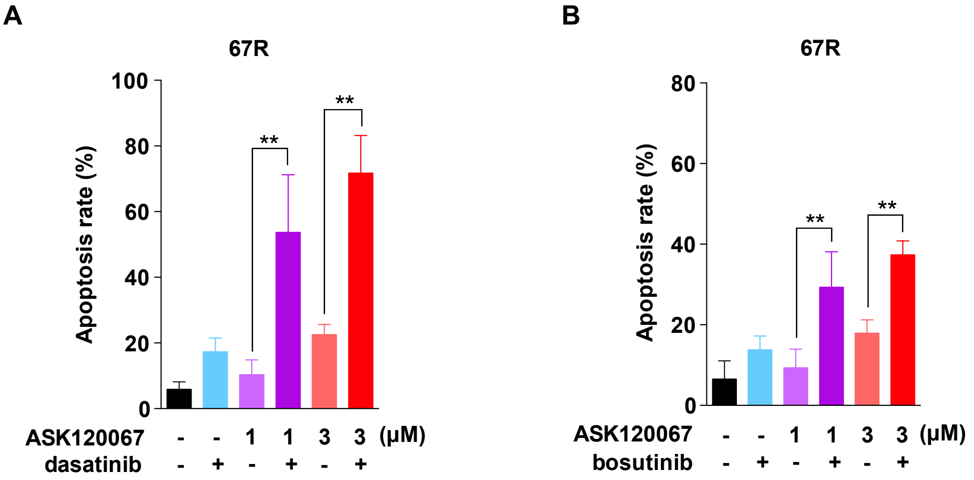

Fig. S5 Combination of ASK120067 with either dasatinib (**a**) or bosutinib (**b**) partially restored the apoptosis-inducing activity of ASK120067-resistant cells to ASK120067 treatment. Data are plotted as the mean ± SD, and the significance of differences was evaluated by Student's *t* test (*$p < 0.05$, **$p < 0.01$).

Supplementary Figure 6

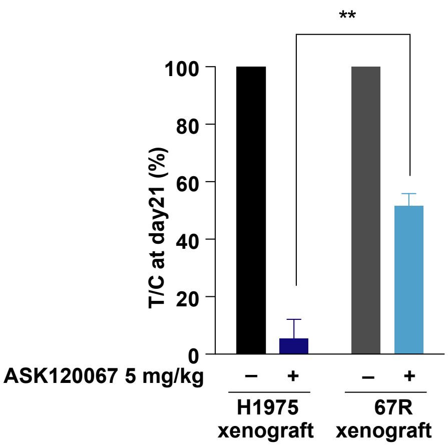

Fig. S6 Comparison of the *in vivo* antitumor efficacy of ASK120067 in an NCI-H1975 xenograft model and ASK120067-resistant xenograft models. Data are plotted as the mean ± SD, and the significance of differences was evaluated by Student's t test (*$p < 0.05$, **$p < 0.01$).
