# Supplementary material for: Discovery of a novel third-generation EGFR inhibitor and identification of a potential combination strategy to overcome resistance
Source: Mol Cancer. 2020 May 13;19:90. doi: 10.1186/s12943-020-01202-9 (PMC7218543; doi:10.1186/s12943-020-01202-9)
Supplement: Supplementary file 2 — Additional file 2 Supplementary materials and methods. [file 12943_2020_1202_MOESM2_ESM.docx]

**Supplementary Materials and Methods**

**Human tyrosine kinase array**

Phosphorylation levels of 71 different tyrosine kinases in parental NCI-H1975 cells or ASK120067-resistant cells (67R) were detected by a Membrane Antibody Array (#AAH-PRTK-1, RayBiotech). All procedures were performed according to the manufacturer's instructions.

**shRNA-mediated knockdown**

Lentiviral vectors encoding shRNA for Ack1 (Generay) and packaging plasmids (psPAX2 and Pmd2. G, Addgene) were transfected into 293T cells, and the virus was collected according to the manufacturer’s instructions. Target cells were infected with 6 μg/mL polybrene for 4-6 h. 48 hours after the infection, positive cells were selected with 0.5 μg/mL of puromycin for at least 7 days. The knockdown efficiency was measured by immunoblotting analysis.
